# Hop Extract Acts as an Antioxidant with Antimicrobial Effects against *Propionibacterium* *Acnes* and *Staphylococcus Aureus*

**DOI:** 10.3390/molecules24020223

**Published:** 2019-01-09

**Authors:** Natalja Weber, Klaus Biehler, Kay Schwabe, Birgit Haarhaus, Karl-W. Quirin, Uwe Frank, Christoph M. Schempp, Ute Wölfle

**Affiliations:** 1 Research Centre skinitial, Department of Dermatology, University Medical Center, 79104 Freiburg, Germany; natalja_weber@gmx.de (N.W.); birgit.haarhaus@uniklinik-freiburg.de (B.H.); christoph.schempp@uniklinik-freiburg.de (C.M.S.); 2 Institute for Infection Prevention and Hospital Epidemiology, Medical Center, University of Freiburg, 79106 Freiburg, Germany; klaus.biehler@uniklinik-freiburg.de (K.B.); uwe.frank@uniklinik-freiburg.de (U.F.); 3 BSI-Beauty Science Intelligence GmbH, 30855 Langenhagen, Germany; K.Schwabe@bsi-cosmetics.de; 4 Flavex Naturextrakte GmbH, 66780 Rehlingen, Germany; wq@flavex.com

**Keywords:** acne, *P. acnes*, *S. aureus*, hop extract, antimicrobial activity, antioxidant activity

## Abstract

Acne is associated with hyperkeratosis, elevated levels of skin sebum and growth of *Propionibacterium acnes* (*P. acnes*) and *Staphylococcus aureus* (*S. aureus*). Furthermore, *P. acnes* promotes inflammation by inducing IL-6 production and oxidative stress. The aim of this study was to assess the antioxidant, anti-inflammatory and antibacterial potential of a hop-CO_2_-extract with 50% humulone and lupulone. The susceptibility of *P. acnes* and *S. aureus* to the hop extract was tested by using the broth microdilution technique. The minimal inhibitory concentrations (MIC) for *P. acnes* and *S. aureus* were 3.1 and 9.4 µg/mL, respectively. In addition, the hop extract showed an antioxidative effect with a half maximal inhibitory concentration (IC_50_) of 29.43 µg/mL as well as additional anti-inflammatory effects by reducing the IL-6 expression (IC_50_: 0.8 µg/mL). In addition, a gel formulation with 0.3% hop extract (*w*/*w*) had antibacterial activity against *P. acnes* and *S. aureus* (inhibition zone value: 5.5 mm and 3 mm, respectively) which was significantly superior to the placebo gel. The positive control (a gel with the antibiotic clindamycin) showed an inhibition zone of 9 mm. Due to its antioxidant, anti-inflammatory and antibacterial effects hop extract might be a treatment option for acne-prone skin.

## 1. Introduction

Acne is the most common inflammatory skin disease from which 70–95% of all teenagers temporarily suffer. In about 19% acne continues also to adulthood [1]. Acne is characterized by hyperactivity of sebaceous glands. Increased sebum production (seborrhea) is triggered by a transient hormonal imbalance in favour of testosterone. Principally, acne shows an epidermal hyperproliferation that causes follicular hyperkeratosis (comedones) and perifollicular inflammation (papules and pustules). The most important pathogen linked to acne-prone skin is *Propionibacterium acnes* (*P. acnes*) [2]. *P. acnes* is a Gram-positive, anaerobic, immobile bacterium that populates skin pores and hair follicles. It grows on sebaceous, greasy skin and uses sebum as nutrient source [3]. Sebum plays a role in the pathogenesis of acne [4], because *P. acnes* releases lipases, proteases and hydrolases into the sebum which promotes oxidative stress, inflammation and tissue destruction [5]. Degraded hyaluronic acid can activate the Toll Like Receptor-2 (TLR-2) on follicular keratinocytes eventually leading to the production of pro-inflammatory cytokines (e.g., IL-6, IL-1, TNF-α, or IL-8) [6,7]. On macrophages TLR-2 activation promotes the expression of IL-8 and IL-12 which stimulate hyperkeratinisation, inflammation and oxidative stress [3,8].

Previous studies have shown that *P. acnes* stimulates keratinocyte proliferation by activating the insulin-like growth factor 1 (IGF-1) receptor system [5]. Therefore, degreasing the skin is one treatment option for acne. Recently we were able to demonstrate that a face cleanser with hop extract has a mild and constant degreasing effect with excellent skin tolerability [9]. Xanthohumol and bitter acids (α-bitter acids like humulone and β-bitter acids like lupulone) from hop (*Humulus lupulus* L.) show antibacterial effects against *P. acnes* [10,11].

Besides *P. acnes* also aerobic bacteria such as the skin commensal *Staphylococcus aureus* (*S. aureus*) are increased in their number in acne lesions [3,12]. *S. aureus* is a harmless Gram-positive coccus that populates skin and mucosa [13]. However, it can also cause inflammatory skin diseases with pustules (e.g., furuncles, abscesses and folliculitis) by the release of extracellular toxins and enzymes.

This is why antibiotics are widely used to treat acne. The use of antibiotics, however, may promote the emergence and spread of bacterial resistance. Resistant *P. acnes* strains have been described more than forty years ago [14]. Resistance of *P. acnes* to clindamycin, tetracycline and other antibiotics is increasing worldwide [8]. On average, about 50% of *P. acnes* strains have been reported as resistant against several antibiotics [14,15].

Methicillin resistant strains of *S. aureus* (MRSA) pose a challenge in the treatment of infections. Resistance arises usually by the acquisition of a gene encoding a penicillin-binding protein (PBP2a) with significantly lower affinity for β-lactams [16].

Therefore, new antiseptic and antimicrobial agents for the topical treatment of skin infections are needed. Plant extracts might serve as alternative treatment options for acne [3].

In this study, we tested a hop extract rich in humulones and lupulones for its antioxidant and anti-inflammatory effects in human primary keratinocytes (HPKs) and, in addition, we analysed its antibacterial properties. In this process we found antimicrobial activities against both *P. acnes* and *S. aureus* (including MRSA) and were also able to demonstrate that the gel formulation containing hop extract unfolds antibacterial activity superior to that of the placebo gel.

## 2. Results

### 2.1. Antioxidant and Anti-Inflammatory Effect of Hop Extract

**Figure 1:**
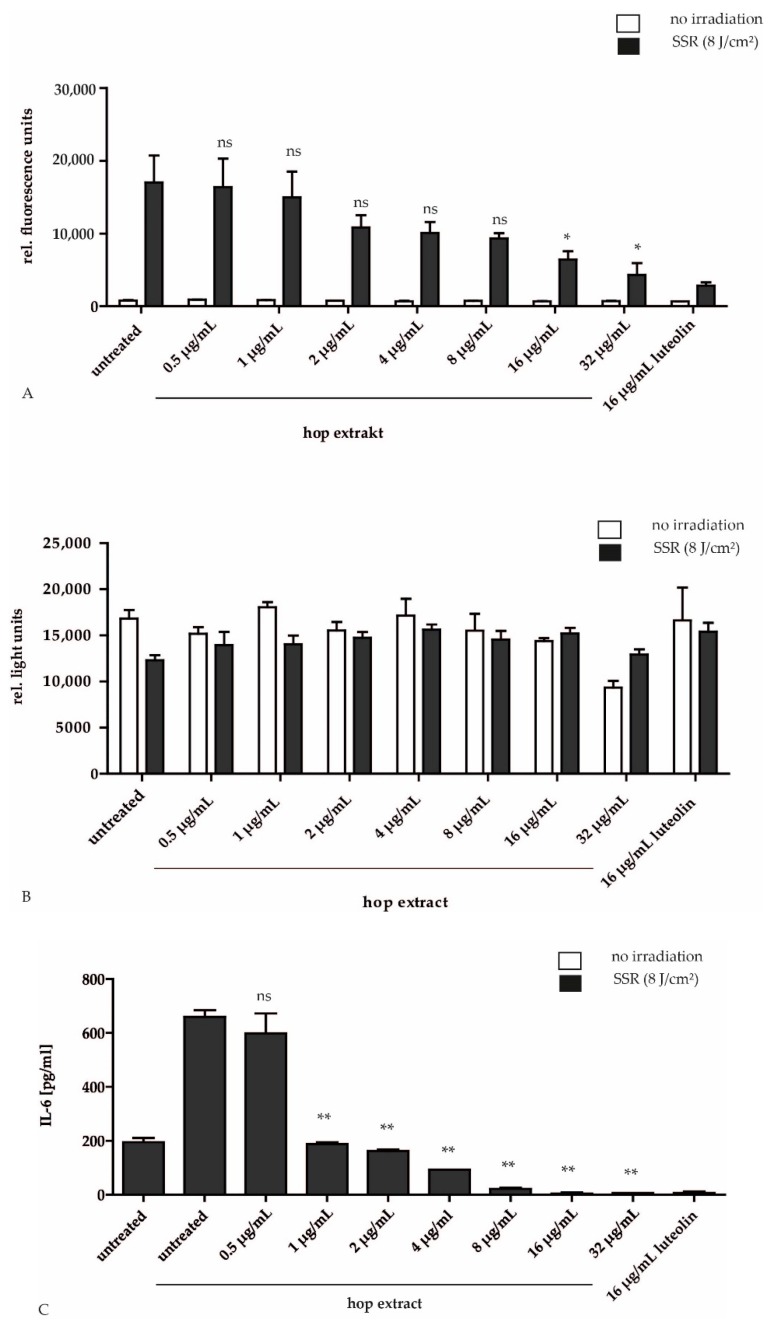
Effect of hop extract on irradiation-induced ROS level and cell viability in HPKs. HPKs were incubated for 30 min with different concentrations of hop extract as indicated or 16 µg/mL luteolin as positive control before CM-H_2_DCFDA was added. (**A**). A test of the cell viability showed that the hop extract was not toxic or phototoxic to the cells (**B**). HPKs were irradiated using a solar simulator and incubated for 24 h with hop extract at various concentrations as indicated. Then the IL-6 concentration of the supernatant was measured with an ELISA. Hop extract reduced irradiation-induced IL-6 production (**C**). Data are expressed as means ± SD of three independent experiments (ns, not significant, *p* ≤ 0.05 (*) and *p* ≤ 0.01 (**).

Acne lesions contain a high Reactive Oxygen Species (ROS) and high pro-cytokine level. To test the antioxidant effect of hop extract we used as test system irradiated HPKs, because ultraviolet (UV) radiation induces extensive generation of ROS in the skin. The ROS scavenging activity of hop extract was determined by using the free radical sensitive fluorescent dye CM-H_2_DCFDA in solar simulator-irradiated HPKs. We chose an irradiation dose of 8 J/cm² in all experiments because this dose induced pronounced ROS production without cytotoxic effects. We could demonstrate that hop extract reduced the formation of ROS-induced dichlorofluorescein (DCF) in a concentration-dependent manner, starting at a concentration of 2 µg/mL. At a concentration of 32 µg/mL the effect was comparable to the potent antioxidant flavonoid luteolin (Figure 1A). The half maximal inhibitory concentration (IC_50_) of hop extract was 29.43 µg/mL. The reduced metabolic activity of HPKs caused by irradiation was rescued with 0.5 µg/mL hop extract. Furthermore, the hop extract was not toxic or phototoxic within the tested concentration range. Only at the highest tested concentration (32 µg/mL) a slight inhibition of the metabolic activity could be detected (Figure 1B). Hop extract also reduced IL-6 production after solar simulator-irradiation of HPKs (IC_50_: 0.8 µg/mL) (Figure 1C). The effect was even stronger compared to the positive control luteolin.

### 2.2. Antibacterial Effect of Hop Extract

*P. acnes* colonization is a relevant factor causing hyperkeratosis and inflammation in acne pathogenesis. The antibacterial effect of hop extract was analysed in a microdilution test with different bacterial strains of the human skin microbiome. Four different strains of *P. acnes* (human skin commensals that prefer anaerobic growth conditions) and four different strains of *S. aureus* (human skin commensals that grow under aerobic growth conditions) were tested. Clindamycin was used as positive control. Clindamycin belongs to the lincosamide class of antibiotics that block the protein synthesis of anaerobic and aerobic bacteria including *P. acnes* and *S. aureus*. Hop extract inhibited the growth of *P. acnes* with a minimal inhibitory concentration (MIC) of 3.1 µg/mL in all *P. acnes* strains tested, including the standard strain *P. acnes* ATCC 6919. Only *P. acnes 201* showed a MIC between 3.1 and 6.2 µg/mL. The MIC of clindamycin was less than 0.2 µg/mL except for the strain *P. acnes* 1990 with an MIH of 0.8 µg/mL (Table 1). The MIC of hop extract in *S. aureus* strains was on average 9.4 µg/mL (range 6.25 µg/mL to 12.5 µg/mL) including MRSA and non MRSA strains. For clindamycin, the MIC was for all non-MRSA strains on average 0.126 µg/mL (range 0.003 µg/mL to 0.25 µg/mL). Although clindamycin very effectively inhibited the growth of non-MRSA strains it could only inhibit the growth of the MRSA strain 4810 at a concentration of >50 µg/mL. In contrast, hop extract inhibited MRSA already at a concentration of 12.5 µg/mL (Table 2).

### 2.3. Antibacterial Effect of a Botanical gel with Hop Extract

**Figure 2:**
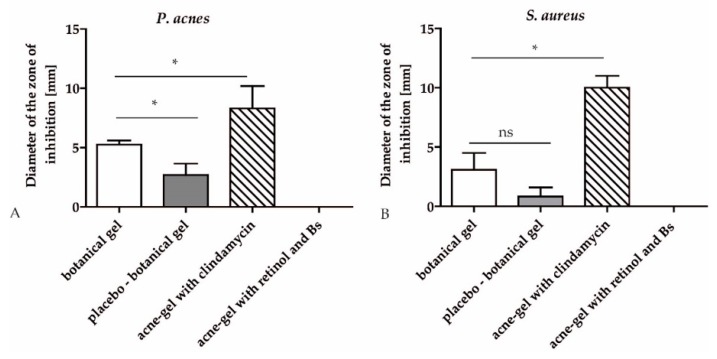
Antibacterial activity of hop extract against *P. acnes* strains determined in the agar diffusion test. Diameter of the zone of inhibition [mm] is indicated. A commercially available acne-gel with clindamycin served as positive control. Another commercial acne-gel with retinol and a *Boswellia serrata* extract (Bs) was additionally tested (**A**). The antibacterial activity of hop extract against *S. aureus* strains was determined in the agar diffusion test. The diameter of the inhibition zone [mm] is indicated. The acne-gel with clindamycin served as positive control. The acne-gel with retinol and a *Boswellia serrata* extract (Bs) was a comparative product (**B**). Data are expressed as means ± SD of two independent experiments (ns, not significant, *p* ≤ 0.05 (*)).

In order to test if the hop extract is also effective in a topical gel preparation, an agar diffusion test was performed. 0.3% hop extract was used in the botanical gel, together with other botanical actives, that is, salicylic acid, *Salix daphnoides* bark extract, *Gentiana lutea* root extract, *Leptospermum scoparium* branch/leaf oil (Manuka oil), Mentha arvensis herb oil and the preservatives sodium levulinate and sodium anisate. The placebo gel contained none of these active ingredients. The botanical gel significantly inhibited the growth of *P. acnes* compared to the placebo gel (4.9 vs. 2.0 mm inhibition zone). The placebo showed also a slight inhibition, possibly due to ingredients such as jojoba oil that also displays moderate antibacterial effects. The positive control was a commercially available gel containing not only clindamycin but also benzoyl peroxide. It inhibited the bacterial growth strongly with an inhibition zone of 9.0 mm. We also tested another commercially available acne gel with retinol and *Boswellia serrata* extract, which showed no antibacterial effect (Figure 2A).

When tested on *S. aureus* the botanical gel also showed a trend to an increased inhibition zone compared to placebo but this effect was not statistically significant. The positive control clindamycin showed an inhibition zone of 2 mm in MRSA while the botanical gel had no effect (Figure 2B). The effect of the positive control was possibly mediated by benzoyl peroxide, because clindamycin alone showed only moderate activity against MRSA in the microdilution test.

## 3. Discussion

The topical standard treatment of acne includes benzoyl peroxide and retinoids as well as topical antibiotics such as clindamycin. Retinol as vitamin A derivative reacts comedolytic and is known for its reduction of keratinocyte mitosis, hyperkeratinisation and inflammation [3]. However, these treatment options have also some disadvantages, because benzoyl peroxide and retinoids on the one hand can irritate the skin and frequently applied topical antibiotics on the other hand may lead to bacterial resistance, so that these antibiotics become useless in severe infections [17]. Therefore, plant extracts are interesting new sources of antibacterial agents. The hop extract used in this study was effective against *P. acnes* and *S. aureus* including MRSA, so that a botanical gel with hop extract might be a useful alternative in the treatment of acne. In a patch test on the back of 30 healthy volunteers the botanical gel did not cause any skin irritation (data not shown).

Good antibacterial activity of isolated compounds and plant extracts are 10 µg/mL and 100 µg/mL, respectively [2,18]. The hop extract has a high antimicrobial activity against *P. acnes* (MIC of 3.1 µg/mL) with additional antioxidant and anti-inflammatory activities as demonstrated in hop-treated irradiated HPKs.

Antibacterial activities of a hop-CO_2_ extract could also be demonstrated against *Corynebacterium xerosis* and *Staphylococcus epidermidis* [19]. Furthermore the growth of other gram-positive bacteria (*Bacillus anthracis, Bacillus subtilis, Corynebacterium diphteriae, Sarcinia lutea, Sarcinia Faecalis, Lactobacillus brevis* including species of *Micrococcus, Mycobacterium*, *Streptomycetes, Listeria and Costridium*) [19,20,21,22,23,24] as well as some gram-negative bacteria (*Helicobacter pylori* and *Brucella* species) could be inhibited by humulone and lupulone [24,25,26]. The isoprenyl side chains of the hop acids interfere with the bacterial cell plasma membrane and cause a leakage of this membrane which inhibits the transport of sugar and amino acids [19,27]. The antibacterial action of hop compounds has also proton ionophore activity and a pronounced redox reactivity, causing cellular oxidative damage. This interference with redox-sensitive pathways is also responsible for antiviral effects of the hop extract. [28].

In addition, especially lupulone and xanthohumulone can penetrate biofilms of *Staphylococcus* species including methicillin resistant strains and reduce the number of bacteria in it. Furthermore lupulone and xanthohumol could also eliminate the bacteria at higher concentrations (~60 μg/mL for xanthohumol and ~125 μg/mL for lupulone) [29].

Many medical plants with anti-inflammatory or antibacterial effects are interesting sources for the treatment of acne [3,30]. When the hop extract is compared to other plant extracts it appears to be very active. Other extracts for example, herbal ball extract (a combination of *Centella asiatica* extract with Kalmegh (*Andrographis paniculata*)), rosmarinic acid, a phenolic compound from *Rosmarinus officinalis*, *Centella asiatica* extract alone, or *Rosa damascena* methanolic extract had a MIC of 31.2 µg/mL, 62.5 µg/mL, 5 mg/mL or 2 mg/mL respectively [2,3,31,32]. Other plant extracts with antibacterial activities at lower concentrations include *Punica granatum* bark extract containing 13% ellagic acid with a MIC of 15.6 against *P. acnes* and 7.8 µg/mL against *S. aureus* [33]. *Angelica anomala* was also very effective against *P. acnes* with a MIC of 15.6 µg/mL [34]. In accordance to our results Yamaguchi and colleagues [11] demonstrated that xanthohumol and lupulones from *Humulus lupulus* possess strong inhibitory activity against *P. acnes* (MIC 0.1–3 µg/mL) [11]. The here tested hop extract possesses also lupulones and humulones but no xanthohumol. In a recent paper by Bartmańska and colleagues it was shown that various polar extracts and isolated flavonoids obtained from spent hop cones also display pronounced antimicrobial effects against a wide range of bacteria including methicillin resistant *S. aureus* [24]. Other polar constituents that occur in hop extracts such as catechin and kaempferol also show antibacterial activities, for example by inhibiting the lipase of *P. acnes* [35] Furthermore Di Sotto and colleagues have shown that hydroalcoholic extracts of hop with phenolic compounds display antiviral and antioxidative effects. [28]

The contribution of *P. acnes* to the pathogenesis of acne also involves the induction of ROS production and release of pro-inflammatory cytokines Sharma et al. described that Echinacea extract normalizes elevated cytokine IL-6 levels comparable to hop extract [36].

Although hop extract is the most important component of the botanical gel tested here, the gel also contains willow bark extract, *Mentha arvensis* oil and *Leptospermum scoparium* oil which display additional useful effects in the topical treatment of acne. Willow balk extract has keratolytic activity and removes cells that plug the secretory duct of sebaceous glands. *Mentha arvensis* oil (field mint oil) with 90% menthol activates the cold-sensing Ca^2+^ permeable non-selective cation channel TRPM8 (Transient Receptor Potential Melastatin 8) [37] and causes a cold sensation and itch relief. *Leptospermum scoparium* oil (Manuka oil) displays additional anti-bacterial activity. Manuka oil inhibited in 40 consecutive aromatogramms the growth of *S. aureus* isolated from patient samples (unpublished data of biovis Diagnostik, MVZ GmbH, Limburg, Germany). The antimicrobial effect of the botanical gel cannot be attributed to a certain substance or extract contained in the gel. Several of the botanical ingredients, that is, hop extract (= *humulus lupulus* extract), salicylic acid, willow birch extract and leptospermium extract certainly may display antimicrobial effects. Therefore, in the agar test the effect of the whole composition was compared to the placebo gel without the botanical ingredients and to other commercially available topical products. This allows the conclusion that all the botanical ingredients together display antimicrobial effects that are superior to the placebo gel.

The acne-gel with retinol and *Boswellia serata* extract had no effect in our agar dilution test. However, it has been shown that *Boswellia serrata* extract is highly effective at low concentrations against aerobic and anaerobic bacteria including *P. acnes* (MIC: 1 µg/mL; [38]) while 11-keto-β-boswellic acid was not active against these bacteria. Therefore, it was speculated that the effective components of Boswellia might rather be essential oils than boswellic acids. The *Boswellia serrata extract* in the tested acne-gel possibly did not contain enough antibacterial substances, or these substances were not released from the acne-gel. One possibility to make substances bioavailable is the preparation of a substance-loaded myristic acid microemulsion. Liu and Huang described such a preparation for curcumin. As curcumin acts as an anti-inflammatory and anti-microbial agent against *P. acnes* and *Staphylococcus epidermidis*, a bacterium that is also involved in acne, the curcumin microemulsion might also be suitable for the treatment of acne [39,40,41]. In the study presented here hop extract obviously could be released from the botanical gel without microemulsions, because in the agar diffusion test the growth of *P. acnes* and *S. aureus* was inhibited effectively.

A review on dermatologically relevant plants to treat acne with focus on controlled clinical studies revealed tea tree oil (*Melaleuca alternifolia*) as one of the most active plant extracts. In a single-blind, randomized study 5% tea tree oil was compared with the gold standard 5% benzoyl peroxide. Both preparations showed after 3 months of treatment comparable improvement of the symptoms [42]. This result was confirmed in a vehicle-controlled randomized double-blind study over 45 days in 60 acne-prone patients [43,44]. Interestingly, tea tree oil is related to manuka oil that is present in our botanical gel with hop extract. In addition, a 2% lotion with green tea (*Camellia sinensis*) extract improved acne symptoms within 6 weeks (application twice daily) in a prospective, non-randomized study with 20 acne patients [44,45,46]. Tannins and flavonoids contained in the green tea extract might be responsible for this anti-acne effect. Flavonoids display antibacterial activity and tannins have anti-inflammatory effects. Epigallocatechin gallate (EGCG) as main active compound in green tea is sebosuppressive and displays antibacterial effects against *P. acnes*. [47]. The phloroglucinol derivatives humulone and lupulone from hop extract might have similar effects.

## 4. Materials and Methods

### 4.1. Chemicals

Clindamycin hydrochlorid (European Pharmacopeia Reference Standard) was purchased by Council of Europe EDQM (Strasbourg, France), the brain heart infusion (BHI) medium by Oxoid (München, Germany), Mueller Hinton (MH) broth, cation adjusted by Becton, Dickinson (Heidelberg, Germany) and Muller Hinton-agar by Roth GmbH (Karlsruhe, Germany).

### 4.2. Hop Extract

**Figure 3:**
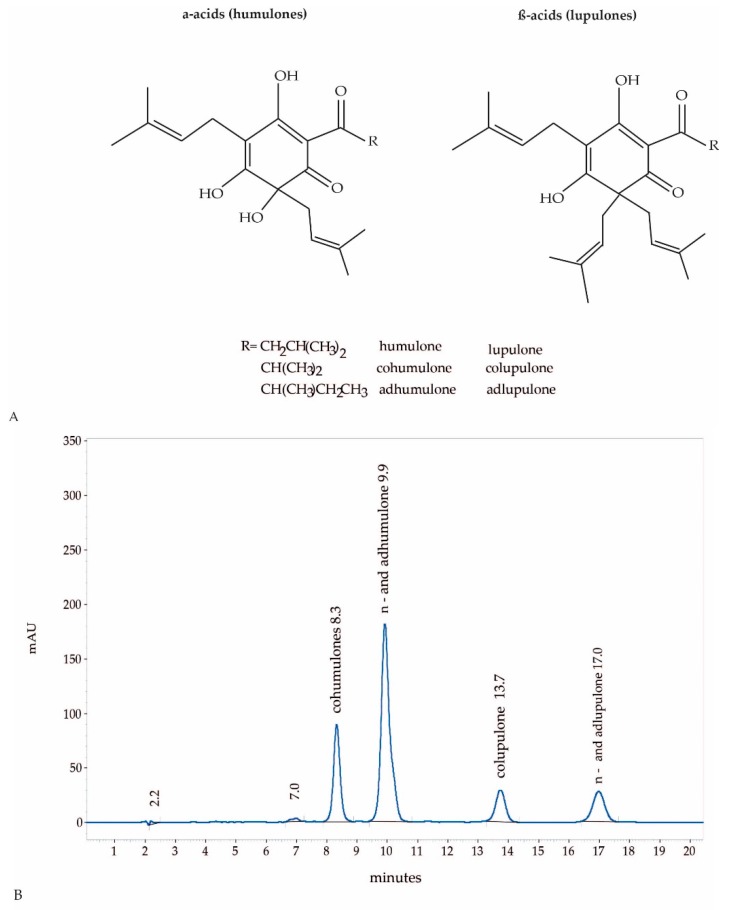
Structural formula of humulones and lupulones (**A**). HPLC fingerprint of hop-CO_2_ extract, flavour reduced and standardised to 50% humulones and lupulones (**B**).

The supercritical hop CO_2_-extract was produced by Flavex (Rehlingen, Germany) and dissolved in DMSO. In brief, hop flowers (*Humulus lupulus* flos) were dried, powdered and extracted with high pressure supercritical CO_2_. The genuine CO_2_ extract is further processed by CO_2_ counter current column extraction in order to remove the essential oil to a large extend. The flavour reduced supercritical extract which does not contain xanthohumol, was standardized to 49–51% humulones (α-acids consisting of humulone, adhumulone and cohumulone) and lupulones (β-acids consisting of n-lupulone, adlupulone and colupulone) by sunflower oil addition [19]. The CO_2_ extract selectively yields lipophilic compounds, therefore no polar compounds such as tannins, flavonoids or other polyphenols are present in the extract. The primary extract is a thick material difficult to handle, thus, sunflower oil is added to obtain a more viscous extract and to standardize the extract to a defined range of active compounds. The ratio of humulones and lupulones may show some seasonal fluctuations. The concentration of humulones varies between 31 und 35% and the concentration of lupulones varies between 15 and 19%. The ratio of humulones to lupulones lies between 1.6 and 2.3 to 1. This indicates that the variations are not very pronounced. This is important as the antibacterial activity of hop acids increases with decreasing polarity. In lupulones compared to humulones a hydroxyl-group is replaced by an isoprenyl side chain. Therefore lupulones are a bit more lipophilic and therefore a bit more active than humulones (Figure 3A).

The final product represents a drug/extract ratio of about 4–5.3/1 and contains less than 2% volatile constituents.

### 4.3. HPLC Analysis of Hop CO_2_-Extract

All solvents were of HPLC grade and supplied by VWR International GmbH (Darmstadt, Germany). International Calibration Extract (ICE) is used as standard and dissolved in methanol with 0.6–1.0 mg/mL concentration. The hop CO_2_-extract was dissolved under sonication in methanol with 0.5–1.5 mg/mL concentration and filtered through a PTFE filter with 0.45 µm pore diameter before injection.

A Merck Hitachi LaChrom Elite HPLC system was used, consisting of auto sampler L-2200, pump L-2130, DAD detector L-2420, column oven L-2350, operated with software EZ Chrom Elite Version 3.3.2 Build 1037 (SP2) from Agilent Technologies, Inc., Santa Clara, CA, USA; column Lichrospher 100 RP-18e (5 µm), 250 × 4 (mm length × internal diameter), Merck KGaA (Darmstadt, Germany). Eluent A was water acidified with o-phosphoric acid 85% to pH 2.3–2.6, eluent B methanol; eluent composition: 14% A, 86% B, isocratic for 20 min. Eluent flow was 1 mL/min, oven temperature 35 °C, detector 314 nm, injection volume 10 µL. Humulones and lupulones were identified and quantified using the Calibration Extract as external standard. A HPLC fingerprint of hop-CO_2_ extract is shown in Figure 3B.

### 4.4. INCI of the Tested Gels

Botanical gel with hop extract (Velan Clear Skin, VELAN skincare, Freiburg, Germany)

INCI: Aqua, Alcohol, Dicaprylyl Ether, Simmondsia Chinensis Seed Oil, Bambusa Arundinacea

Stem Powder, Glycerin, Sodium Stearoyl Glutamate, Hydrogenated Vegetable Glycerides, Salicylic Acid, Salix Daphnoides Bark Extract, Humulus Lupulus Extract, Gentiana Lutea Root Extract, Leptospermum Scoparium Branch/Leaf Oil, Mentha Arvensis Herb Oil, Helianthus Annuus Seed Oil Unsaponifiables, Xanthan Gum, Glyceryl Caprylate, Tocopherol, Cetearyl Alcohol, Sodium Levulinate, Sodium Anisate, Lysolecithin, Sclerotium Gum, Pullulan, Silica, Limonene.

Placebo of the botanical gel (Beauty Science Intelligence GmbH, Langenhagen, Germany)

INCI: Aqua, Alcohol, Dicaprylyl Ether, Simmondsia Chinensis Seed Oil, Bambusa Arundinacea Stem Powder, Sodium Stearoyl Glutamate, Hydrogenated Vegetable Glycerides, Xanthan Gum, Cetearyl Alcohol, Tocopherol, Lysolecithin, Sclerotium Gum, Pullulan, Silica.

Acne-gel with clindamycin (Duac Acne Gel, GlaxoSmithKline GmbH & Co. KG, München, Germany)

INCI: carbomer, dimeticone, disodium lauryl sulfosuccinate, disodium edetate, glycerol, silica (dental type), poloxamer 182, purified water, sodium hydroxide, clindamycin phosphate and benzoyl peroxide.

Acne-gel with Retinol and *Boswellia serrata* (Bs) extract (BiRetix Duo, IFC Dermatologie Deutschland GmbH, Ainring, Germany)

INCI: Aqua, Glycerin, Butylene Glycol, Sodium Acrylate/Sodium Acryloyldimethyl Taurate Copolymer, Polysorbate 80, Dimethyl Isosorbide, Benzyl Alcohol Isohexadecane, Hydrogenated Lecithin, Trehalose, Salicylic Acid, Tocopherol, Hydroxypinacolone retinoate, Phenoxyethanol, Lecithin, Dehydroacetic Acid, Palmitoyl Hydroxypropyltrimonium Amylopectin/Glycerin Crosspolymer, Oryza Sativa Bran Extract, Polysorbate 20, Boswellia Serrata Extract, Honey Extract, Retinol, Ascorbyl PalmitateBeta-sitosterol, Squalane, Glycine Soja Oil, Sodium Hydroxide, Oligopeptide-10, Citric Acid.

### 4.5. Cultivation of Bacteria

In the present study bacteria strains of *P. acnes* and *S. aureus* were analysed. All isolates came from patient isolates of the Institute for Infection Prevention and Hospital Epidemiology, Medical Centre-University of Freiburg (*P. acnes* 199, *P. acnes* 201, *P. acnes* 209) or were purchased by American Type Culture Collections (ATCC; *P. acnes* ATCC 6919 was used as reference strain). Furthermore *S. aureus* ATCC 29213, *S. aureus* ATCC 25923, *S. aureus* 2407 and *S aureus* 4810 (MRSA) were analysed. The test strains were pre-cultivated on appropriate agar plates (MH for *S. aureus*, BHI for *P. acnes*). Fresh colonies were suspended in PBS. The final turbidity was assessed according to that of a 0.5 McFarland Standard.

### 4.6. Microdilution Test

The minimal inhibitory concentration (MIC) of bacterial growth was determined by mixing the bacterial culture with the broth BHI or MH. *P. acnes* were grown in BHI medium under anaerobic conditions using an Anaerobe Container System (GasPak EZ) at 37 °C for 3 days and the turbidity of the bacterial suspension was adjusted to an optical density of 0.14 with PBS to obtain about 1 × 10^8^ colony-forming units (CFU)/mL. *S. aureus* was grown in MH medium under aerobic conditions at 37 °C overnight and the bacteria were adjusted photometrical to 0.08 (10^8^ CFU/mL) in PBS. 50 µL *P. acnes* suspension or 50 µL *S. aureus* suspension were added to 5 mL 2-fold concentrated BHI or MH medium. 100 µL of this suspension (corresponding to 50,000 bacteria) was used per well in a 96 round bottom well plate. 100 µL sterile water with hop extract was added to each well. The hop extract was dissolved in DMSO and serial diluted in a 2-fold dilution series to achieve 9 concentrations from 0.19 µg/mL to 50 µg/mL. Clindamycin served as positive control. DMSO (solvent of the hop extract) and water (solvent of clindamycin) served as negative controls. It was dissolved in distilled water and was used in 2-fold dilution series to achieve 9 concentrations from 32 µg/mL to 0.125 µg/mL. The *P. acnes* samples were then incubated in an anaerobic environment at 37 °C for 24 h. *S aureus* samples were incubated for 16 h in an aerobic environment. The lowest hop concentration required to inhibit bacterial growth was defined as MIC. Each sample dilution was tested in a duplicate manner with biological replicates. The assay was performed according the guidelines of Clinical and Laboratory Standards Institute (CLSI).

### 4.7. Agar Diffusion Test

The antibacterial activity of several gel preparations was evaluated by the agar diffusion test. *P. acnes* was incubated in BHI for 3 days at 37 °C under anaerobic conditions and adjusted to yield approximately 10^4^ CFU/mL PBS. Agar plates were filled with 12 mL of BHI-Agar or MH agar. The agar was inoculated by spreading *P. acnes* or *S. aureus* over the entire agar surface and allowed to dry. Then 4 holes with a diameter of 3 mm were punched aseptically with a cork drill. The gel formulations were placed in the holes respectively. Afterwards the agar plates with *P acnes* were incubated at 37 °C under anaerobic condition for 3–4 days. The agar plates with *S. aureus* were incubated at 37 °C under aerobic condition for 1 day. The gel base (placebo), the botanical gel (containing hop extract) and an acne-gel (containing retinol and *Boswellia serata* extract) were used. A gel with 1% (*w*/*w*) clindamycin and 5% (*w*/*w*) benzoyl peroxide served as positive control. The antibacterial activity was estimated by measuring the diameter of the zone of inhibition that appeared as clear zone. All diffusion tests were performed in two independent experiments and antibacterial activity was expressed as mean ± standard deviation.

### 4.8. Cultivation of Human Primary Keratinocytes (HPKs)

HPKs were prepared from juvenile foreskin or adult skin obtained from dermatological surgeries and cultured according to the method of Rheinwald and Green [48] in DermaLife medium (Cell System, Goisdorf, Germany). All cells were cultured at 37 °C in a humidified atmosphere with 5% CO_2_. The study for the in vitro research with primary keratinocytes was approved by the ethics committee of the University of Freiburg (no.432/18).

### 4.9. Intracellular ROS Measurement

Intracellular reactive oxygen species (ROS) formation was quantified using 5 µM CM-H_2_DCFDA reagent (5-(and-6)-chloromethyl-2′,7′-dichlorodihydrofluorescein diacetate) as described [49]. In brief, after incubation with different substance concentrations from 0.5–32 µg/mL for 30 min, the cells were incubated with a 5 µM CM- H_2_DCFDA solution at 37 °C in the dark and then irradiated with 8 J/cm² using a solar simulator (Model 81192, Oriel, Stratford, CT [50] equipped with a 1000 W Xenon arc lamp, *Model 81192*, Oriel, Stratford, CT, USA). The generation of ROS was measured by the change in fluorescence due to the intracellular production of 2′,7′-dichlorofluorescein (DCF) using excitation and emission wavelengths of 428 nm and 516 nm, respectively, with a spectrophotometer (Sirius HT-TRF microplate reader, BioTek, Bad Friedrichshall, Germany). After ROS measurement the cells were incubated for a further 4 h at 37 °C and the cytotoxicity assay ViaLight Plus ATP assay (Cambrex, Verviers, Belgium) was performed according to the manufacturer’s instructions [51]. The method is based on the bioluminescent measurement of ATP that is present in metabolically active cells. Luciferase catalyses the formation of light from ATP and luciferin. The emitted light intensity is directly proportional to the ATP concentration and is measured with a luminometer (Sirius HT, BioTek, Bad Friedrichshall, Germany).

### 4.10. IL-6 ELISA

HPKs were treated for 30 min with hop extract in the indicated concentrations of 16 µg/mL luteolin as positive control. Subsequently, the cells were irradiated with 8 J/cm² using a solar simulator, washed once with PBS and supplemented with new medium. 24 h post irradiation IL-6 concentrations were analysed in the supernatants by an ELISA (R&D systems, Wiesbaden, Germany) according to the manufacturer’s protocol. Data were expressed as mean ± SD of two experiments.

### 4.11. Statistical Analysis

The data were analysed using the unpaired Student *t*-test (two-tailed) and statistical significance was established at *p* ≤ 0.05 (*) and *p* ≤ 0.01 (**).

## 5. Conclusions

Taken together, hop extract shows not only antibacterial activity against *P. acnes* and *S. aureus* but also has antioxidant and anti-inflammatory effects. This makes it a promising topical ingredient to treat acne-prone skin and to reduce the application of antibiotics in mild forms of acne.

## Figures and Tables

**Table 1:** Antibacterial activity (Minimal inhibitory concentration, MIC) of hop extract against *P. acnes* strains determined in the microdilution test. *P. acnes* strains were isolated from acne lesions or ordered by ATCC.

*P. acnes* Strains	Hop Extract µg/mL	Clindamycin µg/mL
*P. acnes* 199	3.1	0.8
*P. acnes* 201	3.1–6.2	<0.2
*P. acnes* 209	3.1	<0.2
*P. acnes* ATCC 6919	3.1	<0.2

**Table 2:** Antibacterial activity (Minimal inhibitory concentration, MIC) of hop extract against *S. aureus* strains determined in the microdilution test. *S. aureus* were isolated from pyrogenic skin disorders *or* ordered by ATCC.

*S. aureus* Strains	Hop Extract µg/mL	Clindamycin µg/mL
*S. aureus* ATCC 29213	6.25–12.5	0.25
*S. aureus* ATCC 25923	6.25–12.5	<0.125
*S. aureus* 2407	6.25	0.003
*S. aureus* MRSA 4810	12.5	>50
